# The Graded Incomplete Letters Test (GILT): a rapid test to detect cortical visual loss, with UK Biobank implementation

**DOI:** 10.3758/s13428-024-02448-7

**Published:** 2024-06-18

**Authors:** KXX Yong, A Petzold, P Foster, A Young, S Bell, Y Bai, AP Leff, S Crutch, JA Greenwood

**Affiliations:** 1 Queen Square Institute of Neurology, University College London, London, UK; 2 Institute of Ophthalmology, University College London, London, UK; 3 Moorfields Eye Hospital NHS Foundation Trust, London, UK; 4 The National Hospital for Neurology and Neurosurgery, Queen Square, London, WC1N 3BG UK; 5 Neuro-ophthalmology Expertise Centre, Amsterdam UMC, Amsterdam, NL The Netherlands; 6 Big Data Institute, Nuffield, Department of Population Health, University of Oxford, Oxford, UK; 7 UK Biobank, Stockport, UK; 8 Experimental Psychology, University College London, London, UK

**Keywords:** Posterior cortical atrophy, Alzheimer’s disease, Dementia, Cortical vision loss, Vision testing, Letter recognition, UK Biobank

## Abstract

Impairments of object recognition are core features of neurodegenerative syndromes, in particular posterior cortical atrophy (PCA; the ‘visual-variant Alzheimer’s disease’). These impairments arise from damage to higher-level cortical visual regions and are often missed or misattributed to common ophthalmological conditions. Consequently, diagnosis can be delayed for years with considerable implications for patients. We report a new test for the rapid measurement of cortical visual loss – the Graded Incomplete Letters Test (GILT). The GILT is an optimised psychophysical variation of a test used to diagnose cortical visual impairment, which measures thresholds for recognising letters under levels of increasing visual degradation (decreasing "completeness") in a similar fashion to ophthalmic tests. The GILT was administered to UK Biobank participants (total n=2,359) and participants with neurodegenerative conditions characterised by initial cortical visual (PCA, n=18) or memory loss (typical Alzheimer’s disease, n=9). UK Biobank participants, including both typical adults and those with ophthalmological conditions, were able to recognise letters under low levels of completeness. In contrast, participants with PCA consistently made errors with only modest decreases in completeness. GILT sensitivity to PCA was 83.3% for participants reaching the 80% accuracy cut-off, increasing to 88.9% using alternative cut-offs (60% or 100% accuracy). Specificity values were consistently over 94% when compared to UK Biobank participants without or with documented visual conditions, regardless of accuracy cut-off. These first-release UK Biobank and clinical verification data suggest the GILT has utility in both rapidly detecting visual perceptual losses following posterior cortical damage and differentiating perceptual losses from common eye-related conditions.

**Supplementary information:**

The online version contains supplementary material available at 10.3758/s13428-024-02448-7.

## Introduction

In routine clinical practice, assessments of visual functions are geared towards evaluating the eye. However, you ‘see’ things with your brain. Visual agnosias (from Greek: ‘not knowing’) are the brain-based disorders of perception. Agnosias arise from damage to cortical visual regions following stroke, anoxia or other aetiologies, and are core features of focal neurodegenerative conditions (Devinsky et al., 2008; Hodges & Patterson, 2007; Koedam et al., 2010; Neary et al., 1998; Snowden et al., 2007), in particular posterior cortical atrophy (PCA), the ‘visual-variant of Alzheimer’s’ (Crutch et al., 2017). Distinctions between agnosias, particularly regarding profiles of cortical visual deficits, have been used extensively across research and clinical contexts for over a century (Milner & Cavina-Pratesi, 2018), corroborated by the emergence of subsequent neuroimaging and pathologic investigations (Devinsky et al., 2008; Firth et al., 2019; Hodges & Patterson, 2007). Accurate measurement of these cortical visual deficits is important given their clinical implications (Coslett, 2018), from the need to establish their cause to their use in informing treatment and managing associated disability. It is therefore concerning that these deficits are often overlooked or missed for many years. For example, individuals with PCA typically have multiple appointments with optometrists and ophthalmologists before being referred to neurology or dementia services (Harding et al., 2018; Yong et al., 2023). The average time between symptom onset and formal diagnosis in PCA has been reported as 3-4 years compared to 2 years in late-onset dementia (Chapleau et al., 2024; O’malley et al., 2019), precluding opportunities for timely management and treatment.

The various forms of agnosia can be distinguished based on their impairment of distinct levels of the visual processing hierarchy that subserves object recognition. Agnosias cannot be attributed to deficits at the lowest levels of visual processing, such as diminished visual acuity or field defects. Instead, visual agnosias have been attributed to failures of object recognition at two higher levels – (ap)perceptive and associative (Shallice & Jackson, 1988). Apperceptive agnosia broadly reflects visual degradation, with impaired integration of form and feature information precluding the conscious perception of objects and scenes. Associative agnosia instead reflects impaired access to or degradation of semantic knowledge, with a loss of meaning associated with the object representation despite the preserved ability to consciously perceive objects. For example, someone with apperceptive agnosia may have difficulty matching objects presented from different angles and misperceive visual features (e.g. responding ‘wheel’/‘wire’ to a photograph of a whisk), yet recognise the object based on tactile or verbal cues. Someone with associative agnosia may be able to match such objects but mislabel them as semantically related but visually dissimilar objects (e.g. reporting ‘spoon’ instead of whisk). Within this distinction, core features of PCA fall into the apperceptive category, while associative agnosia may arise in semantic dementia despite otherwise intact perception.

PCA is characterised by predominant cortical visual loss, relative to memory loss, and is considered the most common atypical Alzheimer’s Disease (AD) clinical phenotype, comprising ~10% of AD patients at specialist centres (Graff-Radford et al., 2021). While most commonly underpinned by AD pathology, the PCA syndrome can also arise from Lewy body pathology, and rarely frontotemporal lobar degeneration with tau or TDP-43 inclusions and other neurodegenerative disease (Yong et al., 2023). Visual deficits associated with PCA include an elevation in visual crowding (Yong et al., 2014) – the disruptive effect of clutter on object recognition (Whitney & Levi, 2011), deficits in figure-ground segmentation and shape discrimination, simultanagnosia (an inability to perceive multiple objects at once), and “partonomic” errors (where local features are identified at the expense of the global object/form), amongst others (Lehmann et al., 2011). The age of onset of PCA is typically earlier than other forms of AD, around 50-65 years (Schott & Crutch, 2019). As the disease progresses, symptoms in the various subtypes of AD increasingly converge – those with PCA acquire memory and linguistic difficulties, while those with typical AD can similarly acquire visual deficits in addition to their earlier memory, executive and language deficits.

Various tests have been used to evaluate, screen and diagnose cortical visual deficits (James et al., 2001; Mioshi et al., 2006; Warrington et al., 1991) arising from traumatic brain injury, structural damage to the brain (cancer, metastatic disease), stroke (Lopes et al., 2021) or neurodegenerative conditions such as PCA. These tests often use visually degraded or ambiguous conditions like unconventional orientations, silhouettes, and overlapping or ‘fragmented’ formats to evaluate deficits in object perception corresponding to the apperceptive level described above (James et al., 2001; Mioshi et al., 2006; Riddoch et al., 1995; Torfs et al., 2014; Warrington et al., 1991). Amongst these, a frequently used diagnostic test of object recognition under visually degraded conditions is the recognition of incomplete letters, a subtest of the Visual Object and Space Perception battery (Warrington, 1982). Impairments in incomplete letter recognition have been well-documented following brain lesions and neurodegenerative disease. Accordingly, incomplete letter stimuli are frequently used in standard measures within dementia clinical and research settings (James et al., 2001; Mioshi et al., 2006; Riddoch et al., 1995; Warrington et al., 1991), including in the diagnosis of PCA (Bowen et al., 2019; Schott & Crutch, 2019; Yong et al., 2022). Incomplete letter recognition dissociates with various lower-level deficits, including visual field defects and diminished figure-ground and shape discrimination (Lehmann et al., 2011; McCarthy & Warrington, 1990; Warrington & James, 1988), but has been associated with the increases in visual crowding (Strappini et al., 2017) that occur with PCA. Common clinical manifestations of difficulty with degraded letter forms include struggling to read digital signs or clocks and difficulty recognising fragmented visual test stimuli (e.g. failing on dotted Ishihara test plates despite unaffected colour vision) (Yong et al., 2023). In the context of the dual theory of visual streams, while such deficits are often considered ‘ventral’ in nature (the ‘what?’ stream), impaired performance on such tasks has also been documented in patients with right parietal lesions (McCarthy & Warrington, 1990; Warrington, 1982; Warrington & James, 1967, 1988) overlapping with ‘dorsal’ functions (Milner & Cavina-Pratesi, 2018) commonly referred to as the ‘where/how?’ stream (though for a critical review of the two systems theory, see (Rossetti et al., 2017)). Incomplete letter recognition is estimated to become abnormal early on in PCA and at intermediate stages of typical AD (Firth et al., 2019), consistent with the general convergence of symptoms over time in AD, as above. Impairments on incomplete letters in typical AD are associated with a younger age at onset, likely owing to parieto-occipital atrophy (Pavisic et al., 2017; Smits et al., 2012). Notably, the Incomplete Letters Test has been recommended by eye and neurology professionals to distinguish ocular/optic deficits from cortical visual deficits (Bowen et al., 2019).

Current versions of incomplete letter tasks are however limited in a number of ways, especially in dementia clinical and research settings. Firstly, a limitation of the current version of the test is that letters are presented only with a single level of degradation (predominantly at 30% completeness (Warrington et al., 1991)), with performance measured as percent-correct recognition. This creates a susceptibility to ceiling effects (Firth et al., 2019) – a general limitation of many routine visual measures (Bellio et al., 2020) – and limits the sensitivity of the test in tracking disease progression. Secondly, their use in clinical practice is essentially restricted to highly specialised professionals in neuropsychology, neurology and neuro-ophthalmology, while people with PCA are most commonly initially seen by eye health professionals. In research settings, their use is often restricted to specialised test batteries, with generic batteries featuring few, if any visual measures (Bellio et al., 2020). Thirdly, in the context of neurodegenerative disease, there is mixed evidence regarding the disease-specificity of the impairments on this test. Mixed findings of impaired incomplete letter recognition reflecting AD pathology (Boyd et al., 2014) or mixed pathology (Salmon et al., 2022) have prompted recommendations to better differentiate neurodegenerative conditions, particularly PCA, by evaluating both intact and incomplete letter recognition. Finally, outside professional recommendations (Bowen et al., 2019), there is limited empirical evidence on whether these tests can differentiate cortical from ocular visual deficits (like glaucoma), which are also prevalent in older adults.

Given these gaps in test sensitivity, specificity and utility, and following patient and professional consultation to improve the diagnosis of cortical vision loss (Bowen et al., 2019), we present a novel variation of this test, the Graded Incomplete Letters Test (GILT), to rapidly detect cortical vision loss in agnosia and neurodegenerative conditions. We present preliminary normative data from the UK Biobank re-imaging study (Foster et al., 2023) and compare patients with predominant cortical visual (PCA) or memory loss (typical AD) arising from neurodegenerative disease. The test optimises assessment through psychophysical techniques to measure thresholds for the identification of letters affected by visual degradation (or incompleteness). Rather than tests measuring thresholds using letters under varying contrast or brightness, the GILT uses letters which become progressively less complete on a digital interface. The test is designed to be short (<3 minutes), to minimise ceiling/floor, order and letter effects and to enable the sensitive detection of cortical visual abnormalities.

## Method

### Participants

Participants from UK Biobank (UKB; n=2,359) and the UCL Dementia Research Centre (n=27; 18 with PCA and 9 with typical AD) were administered the GILT. UKB volunteers were administered a version using a touchscreen at Biobank visits (GILT-UKB), while UCL patient participants were administered the test using a portable laptop for home testing.

See Table 1 for participant demographic and clinical information. A number of UKB volunteers had documented conditions which may affect vision (cataract n=109; amblyopia n=88; glaucoma n=60; stroke n=21; low vision [<6/12 acuity] n=4). UCL patients had varying degrees of cortical visual loss which could not be attributed to ophthalmological conditions, stroke or tumour, consistent with clinical diagnoses.

**Table 1:** Total UK Biobank and UCL sample demographic and clinical information

Sample	GILT-UKB (n=2,359)	GILT (n=27)	
	UK Biobank	UCL	
Diagnoses	Cataract n=109; Amblyopia n=88; Glaucoma n=60; Stroke n=21*	PCA n=18	Typical AD n=9
Age (years)	67.0 (61.0, 72.0)	70.6 (63.6, 74.0)	64.6 (61.0, 71.5)
Sex (male:female)	1190:1169	11:7	3:6
β-Amyloid PET/ CSF consistent with AD	-	9/9	7/7
MMSE	-	21.5 (18.0, 25.0)	26.5 (19.5, 29.0)

Medians and interquartile ranges are reported for age and MMSE. CSF: Cerebrospinal fluid, MMSE: Mini-mental state examination. *Unique n without diagnoses or low vision =2,094

UK Biobank: UKB is a population-based prospective cohort study of >500,000 volunteers aged 40-69 years recruited between 2006-2010 (https://www.ukbiobank.ac.uk). Participants completed a touchscreen questionnaire, cognitive testing, verbal interview, and physical examination and provided biological samples. Ethics Committee approval for UKB was obtained from the North West Multi-Centre Research Ethics Committee (Research Ethics Committee reference: 16/NW/0274). The GILT-UKB was administered to volunteers within the UK Biobank Imaging sub-study; the first release data are presented here.

UCL Dementia Research Centre: participants had a diagnosis of PCA or typical AD, and fulfilled consensus criteria for PCA (17 PCA-pure, 1 PCA-plus) and research criteria for probable AD respectively (Crutch et al., 2017; Dubois et al., 2014; McKhann et al., 2011). PCA and typical AD participants were of comparable age; all available molecular pathology was consistent with AD pathology (Table 1). Cortical visual impairments were evident in all PCA patients with relative sparing of memory, language, attention and executive functions. The typical AD group included participants with mild, prodromal AD; correspondingly, clinical severity, cognitive and cortical visual impairments were comparable to or less evident than in the PCA group (Supplementary Table 1). Prior ethical approval for the study was provided by the National Research Ethics Service Committee London Queen Square and informed consent obtained from all participants according to the Declaration of Helsinki.

### Stimuli and procedures

**Fig. 1:**
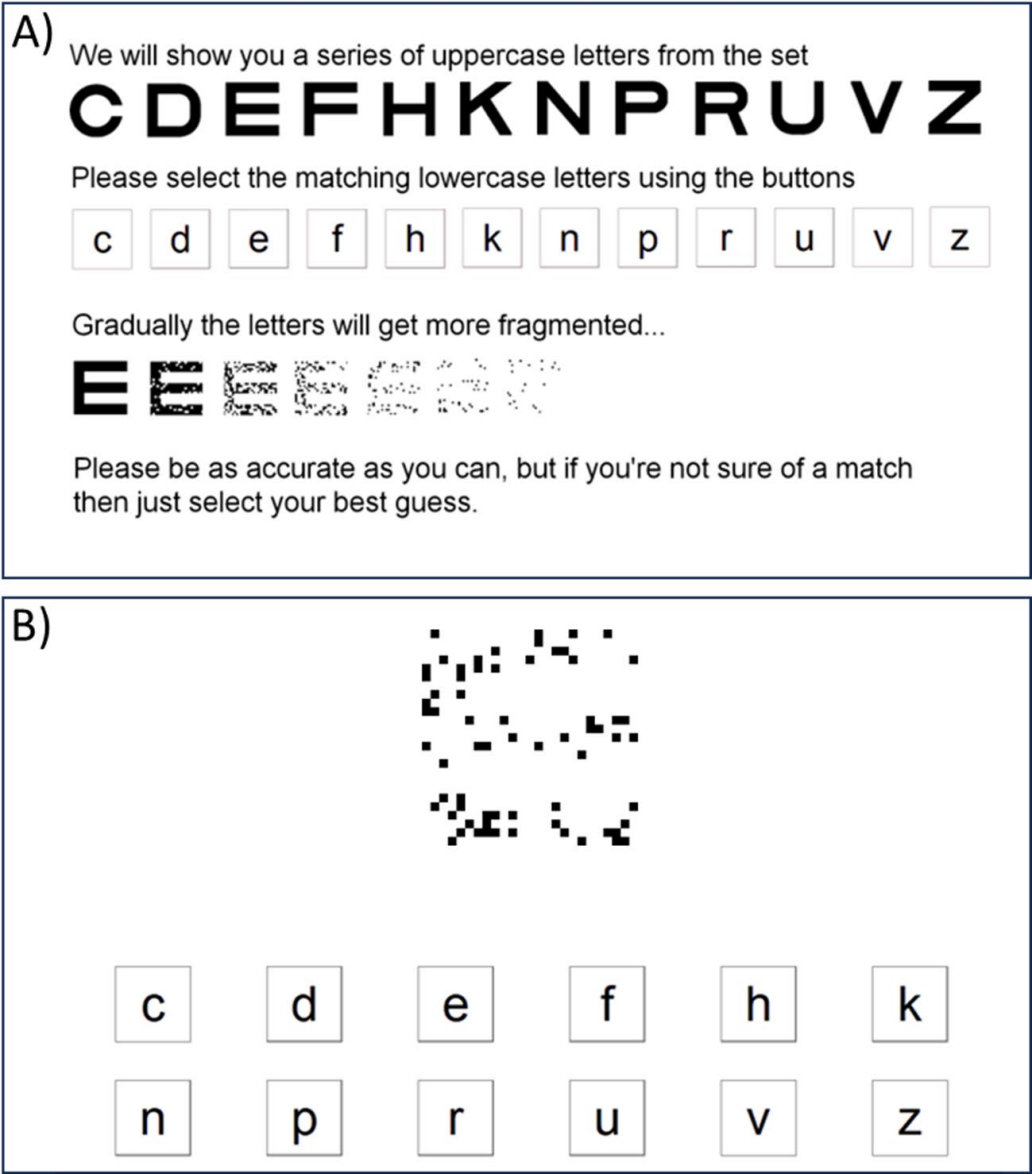
**A**) The GILT instructions screen from the UK Biobank, **B**) an example trial with a single uppercase target (a letter E at 0.159 completeness) and the lowercase response items shown below (target letter: E)

The Graded Incomplete Letters Test was developed from the Incomplete Letters subtest from the Visual Object and Space Perception Battery (VOSP) (Warrington et al., 1991). The VOSP subtest involves the identification of 20 black letters on a white background, visually degraded via random blocks of fragmentation (white sections removed from the black letter) with a fixed black:white ratio of 30:70. The GILT optimises the sensitivity of this test to detect cortical visual abnormalities by adding a range of completeness levels, where ‘completeness’ is defined as the proportion of pixels from a given letter that remain visible in black against the white background (ranging from the whole letter at a completeness of 1 to a completely invisible/absent letter at 0). Letters at different completeness levels are presented using a modified method of limits procedure with forced-choice responses. For each trial, participants were presented with a target letter, which progressively decreased in completeness across trials, and were asked to select the response letter that matched the target in each case. See Figure 1 for example instructions and a trial from the version featured within the UKB study (GILT-UKB).

In detail, all stimuli were presented binocularly from a typical viewing distance (approximately 50cm). On each trial, a single uppercase target letter was presented. This item-by-item presentation was selected over a chart format (as in standard visual acuity or contrast sensitivity assessment) to reduce disruption caused by adjacent letters (crowding) (Song et al., 2014), a particular problem for individuals with cortical visual deficits in whom these effects can be elevated (Yong et al., 2023). Target letters were presented in the Sloan font (Sloan, 1959). This font is a standard for acuity testing due to the fixed proportions of the letters – the stroke width is one fifth the letter diameter and matched for all letter features. In this way, the effect of degradation on the features within letters was equated across the various letterforms. Letters were presented with a diameter of 275 pixels, approximately 8.3 degrees of visual angle (100 minutes of arc per letter stroke). Given this corresponds to logMAR 2.0 (Snellen equivalent: 20/2000), acuity limitations on performance would be unlikely. Letters were black-on-white at 100% Weber contrast, further ensuring that any contrast sensitivity losses were unlikely to limit performance.

On each trial, the target was one of 12 distinct uppercase letters (C, D, E, F, H, K, N, P, R, U, V, Z). These letters were selected to be the same as in the UKB visual acuity testing, based on a standard logMAR chart. Note that this set is expanded from the original Sloan letters, similar to other expanded sets (Shah et al., 2011). Participants were asked to indicate which of these 12 uppercase letters is presented on each trial using a set of lowercase response items (Figure 1). This forced-choice response has a sufficient number of options to minimise the impact of correct guesses on the threshold estimates (Carkeet, 2001), whilst also allowing for response options to be presented to participants simply (compared with the use of the full alphabet, for instance). Response options were presented to participants as two rows of six lower case letters, presented at the bottom of a touchscreen (ELO 1715L 17”, 1280x1024). Target and response letters used differing case and font to preclude strategies relying on letter matching rather than recognition. UCL Dementia Research Centre participants were administered the GILT-UKB with the following adjustments to allow for patient in-home assessments: the task was administered using a portable laptop (Dell Latitude 5500 16”, 1280x1024; presenting stimuli at comparable diameter of approximately 7.8 degrees of visual angle) on which the tester used the touchscreen to register patient verbal responses.

Targets were presented under a total of nine completeness levels, starting with complete letters (a completeness of 1.0) and decreasing with 8 subsequent log-spaced levels (each separated by 0.25 log units to give the following proportions of letter completeness: 0.891, 0.501, 0.282, 0.159, 0.089, 0.050, 0.028, and 0.016). In this way, six of nine levels were of lower completeness than the standard VOSP items (fixed at 0.30 complete). The incremental fragmentation (Figure 1A) allowed for efficient measurement of letter-identification thresholds, whereby each participant quickly approached the level of completeness sufficient to induce errors, consistent with the design of other tests of visual function (e.g. acuity and Pelli-Robson contrast charts (Bailey & Lovie, 1976; Pelli et al., 1988)). As above, the proportion of completeness corresponded to the number of black pixels within each letter divided by the number of black pixels within the complete letter image. Letters of varying completeness were generated using the following steps. Firstly, random noise images were generated with the same size as the letter image (275×275 pixels) with a Gaussian distribution of grey levels. Greyscale noise images were binarized to be black or white ‘checks’, and scaled up such that each check within the noise image was one fifth the size of the letter stroke (i.e. each stroke was the width of 5 checks). The mask for each letter was then applied, such that checks outside the letter boundary were removed. By shifting the mean luminance of the greyscale noise image prior to binarization, different levels of completeness could be achieved. This process was repeated iteratively until the final image reached the desired level of completeness.

Trials were presented with a modified method of limits procedure, beginning at full completeness (i.e. 1) and decreasing, again following a similar design to other visual tests (Bailey & Lovie, 1976; Pelli et al., 1988). Each completeness level was presented in blocks of 5 trials (similar to the 5-letter lines in acuity charts), for a maximum of 45 trials per participant (5 targets under up to nine completeness levels). The task was discontinued when participants reach a pre-specified accuracy level within the current completeness level. For the GILT-UKB version, the accuracy cut-off was taken at 60% correct (i.e. 3/5 correct within the completeness level) or below. The task was discontinued either at this completeness level, or after a maximum time of 120 seconds after onset of the first trial. For UCL Dementia Research Centre participants, the 60% accuracy cut-off was maintained for consistency, though there was no time limit for the task. To control for stimulus order and letter effects, four testing sets with distinct check patterns and pseudorandomised letter order were randomly assigned to each participant. For each testing set, target letters were randomly assigned to each of the 5-letter blocks (i.e. the letters at each completeness level). Both letter order (within block) and the letters selected within the block (from the 12 possible options) were arranged pseudorandomly so that each letter never appeared twice in consecutive trials, and always appeared in every 20 consecutive trials. Each of the four testing sets was generated as a different image set comprising the above completeness levels, each generated with a distinct distribution of checks.

### GILT measures

We analysed a number of measures to examine the effectiveness of the GILT. We first examined overall performance on the GILT using a broad index of performance, which corresponded to percent correct performance on the letter identification task. These values were calculated across all completed trials and completeness levels.

The primary GILT outcome measures were completeness thresholds for letter identification – the lowest completeness level at which letters can be reliably identified. Threshold measurement requires that this transition (from unseen to seen) be taken at a specific level of accuracy. To determine the best practice for obtaining these thresholds, we compared several measurement approaches.

Cut-off thresholds: A common approach in ophthalmic testing is to score performance “by line” (i.e. each difficulty/completeness level, often denoted by the “lines” in an acuity chart), and to take the threshold as the line at which a desired accuracy level is reached (Sloan, 1959). As above, the GILT-UKB was run with a minimum accuracy cut-off of 60%, meaning that thresholds can be taken as the highest completeness level at which at least 3/5 letters are correctly identified. Because higher thresholds are typically taken in ophthalmic practice (Bailey & Lovie, 1976), we also calculated thresholds with accuracy cut-offs of 100% and 80% - the highest completeness level at which at least 5/5 and 4/5 letters are correctly identified. We refer to these as cut-off thresholds, measured in units of completeness and bounded at levels of 1 and 0.016 (the highest and lowest completeness levels presented).

Letter-based thresholds: One issue with the above cut-off, or “line-based” measurements is that the resolution of the resulting thresholds is limited to the specific difficulty levels tested (here, completeness). A common approach to increase this resolution in visual acuity and contrast sensitivity testing is to use “by letter” scoring (Elliott et al., 1991). Here, responses to each letter contribute to the threshold estimate. Because our completeness levels are log spaced with 0.25 log units of completeness between them, the correct response to each letter can be considered to contribute 0.05 log units of *sensitivity* (the inverse of threshold, since high sensitivity yields a low threshold). Participants start with a sensitivity of 0 and add 0.05 log units with each correct response, excluding the first 5 trials with complete letters (i.e. beginning from 0.891 complete). Scoring is terminated when the accuracy cut-off is reached (which we calculated with 100%, 80% and 60% cut-offs, as above). To compute the threshold *t* from this value of sensitivity *s*, we convert back from the logarithm and take the inverse, with $t=\left(\frac{1}{{10}^{s}}\right)$. We refer to these as letter-based thresholds, bounded at 1 (sensitivity of 0) and 0.01 (40 trials x 0.05 log units= sensitivity of 2).

### Statistical methods

The primary focus of analyses was to determine GILT performance in detecting cortical visual loss and differentiating cortical visual from ocular losses. Analyses report sensitivity, specificity, positive and negative predictive values, observed participant-level data and summary statistics rather than null hypothesis testing. Test statistics are reported when comparing PCA to UKB participants both without and with documented visual conditions, given how eye clinic settings are particularly relevant to detection and differentiation of cortical visual loss. Sample sizes of UKB participants without or with documented visual conditions exceed recommended control samples for neuropsychological studies (McIntosh & Rittmo, 2021). ROC curves were used to investigate the ability of GILT completeness thresholds to differentiate PCA from UKB participants without documented visual conditions (low vision [<6/12 acuity], cataract, glaucoma, amblyopia) or stroke. Discriminatory ability was assessed using logistic regression models relating GILT completeness thresholds to odds of PCA (PCA vs UKB) fitted with Firth's penalized likelihood method to reduce small-sample bias.

## Results

### Overall performance on the GILT: percent correct

**Fig. 2:**
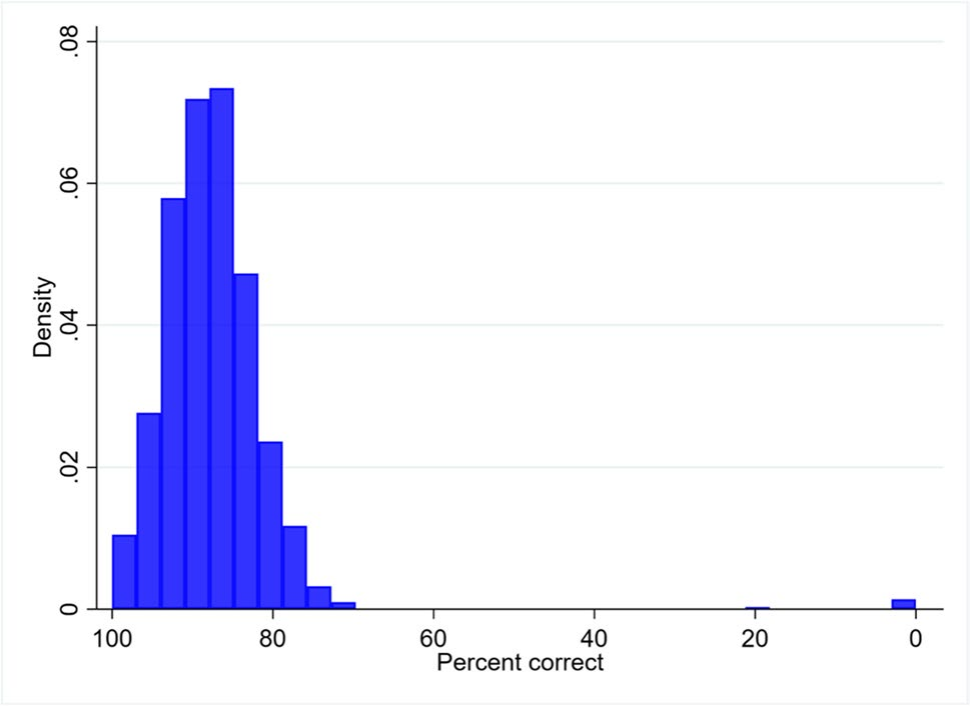
The frequency distribution of GILT percent correct performance in the total UK Biobank sample. Percent correct was calculated across all trials and completeness levels, and is reported for all participants regardless of which accuracy cut-off value was achieved

To give an overview of performance on the GILT, Figure 2 plots the distribution of percent correct performance across all completeness levels in UKB participants, regardless of diagnosis. GILT mean percent correct was 87.3% (SD=8.1) averaged across all UKB participants regardless of diagnosis (n=2,359). Mean percent correct was above 77.5% in 95% of UKB participants, including those with visual conditions (low vision [visual acuity <6/12] and/or presence of cataract, glaucoma, or amblyopia) and stroke. For UCL Dementia Research Centre participants, mean percent correct was 69.4% (SD=9.7) in PCA and 83.4% (SD=3.5) in typical AD participants. Only four of eighteen PCA participants were within the normal range for percent correct based on the total UKB sample (5^th^ percentile: 77.5%), all achieving 80% accuracy (PCA range: 50.0-80.0%). In contrast, all but one typical AD participant was within this normal range (typical AD range: 76.0-86.7%). See Supplementary Figure 1 for further details on percent correct performance in UKB, PCA and typical AD participants.

The majority of participants in UKB reached the specified accuracy cut-offs within the GILT-UKB time limit of 120s; n=2,300 (97%) of UKB participants reached the 100% accuracy cut-off, n=1,967 (83%) reached the 80% accuracy cut-off, and n=1,421 (60%) reached the 60% accuracy cut-off. All PCA and typical AD participants reached the accuracy cut-offs as there was no time limit for administration. For all analyses, between-group comparisons were only made between participants who reached each threshold; in this way, we avoided comparing participants who simply ran out of time at a certain completeness level (i.e. accurate, but slow) with those who reached accuracy cut-offs (i.e 100%; 80%; 60%).

### GILT primary outcome: completeness thresholds

The primary outcome measures for the GILT are completeness thresholds – cut-off or letter-based – for letter recognition (Elliott et al., 1991). Here we report letter-based thresholds using the 80% accuracy cut-off. See Supplementary Figure 2 for comparisons of cut-off and letter-based thresholds at the different accuracy cut-off levels in UKB, PCA and typical AD participants. Cut-off thresholds were restricted to the nine pre-specified completeness levels, while letter-based thresholds were more granular, taking up to 28 values in the UKB sample. Based on these analyses, we report here the letter-based (vs. cut-off based) thresholds owing to their improved resolution compared to cut-off thresholds.

We report thresholds taken at 80% correct performance (i.e. making at least two errors on a given completeness level) rather than 100% or 60% accuracy, for a number of reasons. First, this practice is consistent with the 80% accuracy thresholds commonly used in ophthalmic practice. Second, the higher 100% threshold was considered unsuitable as there are an infinite number of completeness values at 100% accuracy (assuming performance follows the standard sigmoidal function, as is common in psychophysics). Third, the 80% rather than 60% accuracy threshold was preferable as only n=1,421 (60%) of the total UKB sample reached 60% accuracy (making at least 3 errors on a completeness level) within the UKB time limit of 120s, whereas 83% of the UKB sample reached the 80% correct point.

**Fig. 3:**
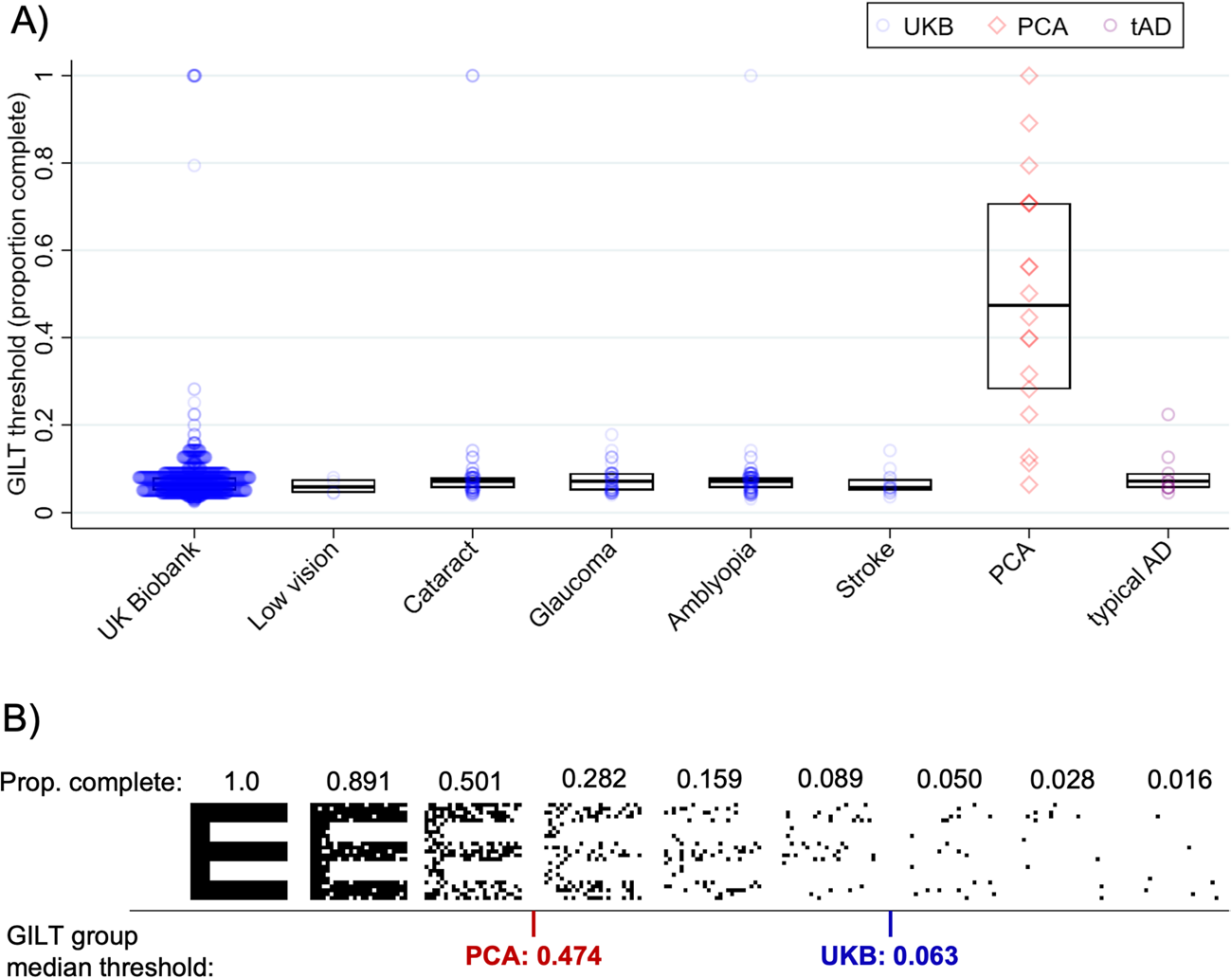
**A**) GILT letter-based thresholds (the proportion completeness of the letters required for accurate letter recognition), with medians and interquartile ranges for UK Biobank (UKB) participants (blue), PCA (red) and typical AD participants (purple). Letter-based thresholds are presented for all UKB participants without or with visual conditions or stroke (as listed on the x-axis) who reached accuracy cut-offs of 80% within the UKB time limit of 120 seconds (total UKB n=1,967). **B**) Example GILT stimuli shown for all of the completeness levels presented for one testing set. Stimuli are presented in relation to GILT median thresholds for PCA (n=18) and UKB groups without or with visual conditions (total n without=1757; with=194). For illustration, threshold values are shown in between the corresponding levels of completeness shown during testing

Figure 3A plots the letter-based thresholds taken as the completeness level required to reach 80% accuracy in the letter identification task. These letter-based thresholds incorporate all of the participants’ responses, with each correct response incrementally contributing until the cut-off threshold of 80% accuracy was reached (unlike line-based/cut-off thresholds, which ignore earlier errors). The thresholds indicate the completeness level that is required for accurate letter recognition (where completeness is expressed as the proportion of the pixels from the full letter that remain visible). In the UCL sample, letter-based thresholds ranged from 6.3-100% in PCA and 4.5-23.4% in typical AD participants. Median thresholds were substantially elevated in individuals with PCA, relative to both typical AD participants and to UKB participants with or without visual conditions. To illustrate the difference in stimulus conditions at these threshold levels, an example set of stimuli is presented in Figure 3B, along with the corresponding median thresholds for the typical UKB sample compared with the PCA individuals.

To consider the sensitivity and specificity of these thresholds, we next defined ‘GILT impairment’ using a routine cut-off value (<5^th^ percentile) based on performance in the UKB sample without documented visual conditions. Table 2 shows the associations between GILT impairment and PCA diagnosis compared to UKB participants, with associated sensitivity, specificity, positive and negative predictive values. Based on these clinical verification data, the GILT shows good sensitivity, with 83.3% of PCA cases correctly identified. The test also shows good specificity with 95.4% of UKB cases correctly rejected as having PCA when compared to UKB participants with common visual conditions (e.g. cataract, glaucoma, amblyopia), and 96.5% correctly rejected when these visual conditions were removed from the UKB sample. While letter-based thresholds at 100% or 60% resulted in increased sensitivity values of 88.9% and comparable specificity values (Supplementary Table 2), letter-based thresholds at 80% accuracy offer more interpretable completeness thresholds while maximizing UKB sample size. Using a penalized likelihood method for small sample sizes, the area under the ROC curve value differentiating PCA from UKB participants without documented visual conditions or stroke was 0.959 using letter-based thresholds at 80% accuracy.

**Table 2:** Associations between PCA diagnosis and GILT impairment, with comparison to UKB either without (left) or with documented visual conditions (right). GILT impairment is defined using a standard cut-off (<5^th^ percentile in UKB without visual conditions) using letter-based thresholds at 80% accuracy. Sensitivity, specificity, positive predictive and negative predictive values (PPV; NPV) are presented. Visual conditions in participants reaching the 80% accuracy cut-off are cataract (*n*=69), amblyopia (n=75), glaucoma (*n*=46) and low vision (*n*=4). UKB participants with stroke reaching 80% accuracy cut-off (*n*=16) have been excluded

	Letter-based threshold: 80% accuracy cut-off			
	PCA diagnosis vs UKB (no visual dx)		PCA diagnosis vs UKB (visual dx)	
	Positive	Negative	Positive	Negative
Positive	15	62	15	9
Negative	3	1695	3	185
Sensitivity	83.3		83.3	
Specificity	96.5		95.4	
PPV	19.5		62.5	
NPV	99.8		98.4	

### GILT completeness thresholds compared to VOSP incomplete letter performance

**Fig. 4:**
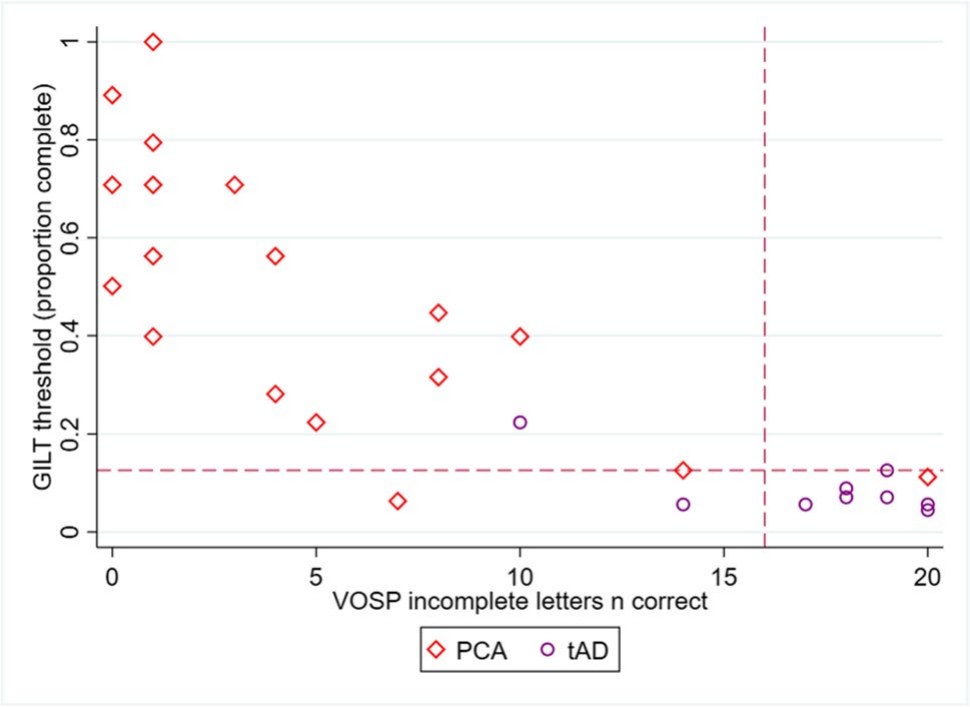
Concordance of GILT letter-based thresholds at 80% accuracy with VOSP incomplete letter accuracy in PCA and typical AD participants. Dashed vertical lines represent the VOSP cut-off indicating impairment (5^th^ percentile: 16; max score: 20 (Warrington et al., 1991)). Dashed horizontal lines represent the GILT 5^th^ percentile cut-off based on UK Biobank participants without documented visual conditions or stroke (5^th^ percentile letter-based threshold: 0.126; max score: 0)

The GILT letter-based thresholds taken at 80% accuracy (as described above) can also be compared with performance on the VOSP incomplete letters subtest, as shown in Figure 4. The VOSP subtest is used in diagnostic settings but features letters at a fixed completeness level (0.30 complete) rather than varying as in the GILT (1, 0.891, 0.501, 0.282, 0.159, 0.089, 0.050, 0.028, 0.016 complete). The granularity of the GILT letter-based thresholds is apparent, with a range of measured GILT performance levels. Broadly, the two measures show the expected association – those who correctly identify the most letters in the VOSP (increasing along the x-axis) also have the lowest thresholds on the GILT (indicating good performance at low completeness levels). Some variation in GILT letter-based thresholds is observed near the lower ends of performance on the VOSP, with PCA patients who show the fewest correct responses on the VOSP exhibiting GILT thresholds between 0.40-1 complete (all above the single 0.30 completeness value of the VOSP letters). Those who performed more highly on the VOSP conversely tended to be associated with low GILT thresholds (indicating good performance), including all of the typical AD participants and two PCA participants.

Most patient participants had concordant impairment on both GILT thresholds and VOSP incomplete letter recognition, defined as performance <5^th^ percentile of UKB participants without documented visual conditions or stroke (for the GILT, as above) or published normative data (VOSP (Warrington et al., 1991)). Using this 5^th^ percentile cut-off, 16/18 PCA patients were considered impaired using letter-based thresholds of 60% or 100% accuracy. Using letter-based thresholds at 80% accuracy, 15/18 PCA and 2/9 typical AD participants exhibited impairment on the GILT compared to 17/18 PCA and 2/9 typical AD participants who exhibited impairment on the VOSP.

## Discussion

The GILT is a rapid digital test optimised to detect subtle cortical visual abnormalities in the form of a difficulty recognising visually degraded letters. Based on normative data from the UK Biobank, we demonstrate in typical adults that the test can ordinarily be performed with a high level of degradation (low ‘completeness’ of the letters). In contrast, PCA participants consistently performed below the normal range of the UK Biobank sample. Despite correctly identifying complete letters, PCA participants exhibited a particular tendency to make errors with decreasing letter completeness, in some cases with very subtle decreases in completeness. This is consistent with the deficits in PCA being specific to stimulus degradation, or low completeness, rather than general letter recognition deficits or other visual issues (e.g. diminished acuity). The same deficits do not manifest in participants with mostly mild, typical AD, whose symptoms primarily concern memory dysfunction. Furthermore, in the UK Biobank, GILT performance was high in both healthy participants and those with ophthalmological visual conditions like glaucoma. In other words, the GILT appears to exhibit a specificity for cortical visual loss arising from PCA-related neurodegeneration. Clinical verification data suggest the GILT may be suitable for differentiating participants with visual loss owing to posterior cortical damage both from typical adult participants and those with common visual conditions.

Our comparison of UK Biobank participants with clinically diagnosed PCA and typical AD participants suggest a number of particular advantages of the GILT and associated letter-based thresholds in research settings. Even with brief test administration (<3 minutes), the GILT provides automatic data capture of measures which are granular while limiting ceiling effects – a particular limitation of existing visual tasks in standard batteries (Bellio et al., 2020), including the Incomplete Letters subtest of the VOSP (Warrington et al., 1991). The increased granularity also increases the sensitivity to abnormalities, including the potential to detect early changes in these abilities and their change over time. Beyond current accuracy and primary outcome measures, analyses of GILT error type (e.g. the extent to which errors relate to the target (Nandy & Tjan, 2007; Yong et al., 2014)) may aid in the differentiation and understanding of abnormal performance. Given that visual symptoms are characteristic initial features of PCA (Firth et al., 2019), GILT thresholds and associated error analyses may have particular promise to detect these early deficits and track the progression of the condition. Beyond PCA, the GILT has promise for detecting early cortical visual losses owing to Lewy body pathology, or non-neurodegenerative aetiology such as encephalitis, head injury, stroke and/or hypoxia (Salmon et al., 2022; Strappini et al., 2017) where visual symptoms occur. We would also expect abnormal GILT performance to emerge at intermediate to later stages of memory- or language-led dementia phenotypes (e.g. typical, amnestic AD or primary progressive aphasia variants (Butts et al., 2015; Firth et al., 2019)) particularly given degeneration of posterior cortical regions.

The UK Biobank sample also allowed us to compare the performance of individuals with other common conditions that affect vision, including low vision, cataract, glaucoma, amblyopia, and stroke. Performance in all of these groups was high, with participants able to recognise letters with low levels of completeness, comparable to typical adults. This distinction is important given that PCA typically emerges at ages around 50-65 years (Schott & Crutch, 2019), where many of these ophthalmological conditions can also become disruptive to vision. There is also a need to exclude the above conditions in diagnostic and research settings because individuals with PCA often present initially to optometrists and ophthalmologists (Harding et al., 2018; Yong et al., 2023) and PCA criteria exclude afferent visual cause (Crutch et al., 2017). The GILT shows great promise in this regard.

Given that cataract, glaucoma, and low vision are associated with reductions in acuity and contrast sensitivity (Christie et al., 2012; Stamper, 1984), the lack of disruption to performance in these groups further suggests that decreased acuity and/or increased blur are not significant limitations on the recognition of incomplete letters, particularly at the large stimulus sizes used in the GILT. Vision in amblyopia is also limited by these factors, as well as an increase in crowding (Greenwood et al., 2012; Levi & Klein, 1985), though these deficits are typically limited to the amblyopic eye. Testing under binocular conditions (as in the UK Biobank) would instead induce suppression of the amblyopic eye (Holopigian et al., 1988), leading performance to be driven predominantly by the better-seeing unaffected eye. It remains possible then that the elevated thresholds of participants with PCA were driven by the elevations in crowding associated with the condition (Yong et al., 2014; Strappini et al., 2017). Higher-level issues with figure-ground segmentation, global integration, and shape discrimination in PCA (Lehmann et al., 2011) are also likely to be limiting factors.

Regardless of their precise origin, it is clear that the test picks up cortical limitations on vision, and that limitations from disruptions to memory and executive function in typical AD do not impair performance. Neuroimaging investigations to assess relationships between GILT measures and the integrity of visual cortical regions will be useful in this regard to further understand the neuroanatomical locus of these abilities. While parieto-occipital atrophy is a key candidate locus, there is evidence that the integration of simple visual features (e.g. contour integration) may be subserved by processes occurring as early as V1 (Gilad et al., 2017). Crowding effects also have neural correlates in V1 (Kwon et al., 2014; Millin et al., 2014) though with modulations that increase throughout the visual hierarchy to V4 (Anderson et al., 2012; Henry & Kohn, 2022) and likely beyond. The neural basis for incomplete letter recognition and associated deficits may similarly derive from multiple levels of processing. Ongoing work in the UK Biobank imaging sub-study and clinic-based research cohort studies is investigating brain-behaviour relationships in the context of degeneration and altered connectivity of visual cortical pathways. Further validation of the GILT is also required incorporating clinico-radiological and biomarker measures. Behavioural investigations that adjust stimulus properties (e.g. stimulus/check size, blur) will also help to determine the mechanisms underpinning abnormal performance.

Amongst the typical UK Biobank population, the GILT produced a small proportion of false positive results, with a further small subset found to make errors with complete letters (0.4% of current UK Biobank sample). However, as a proportion of people within this age range may be amyloid positive and relatedly exhibiting subtle cognitive deficits (Lane et al., 2019; Lu et al., 2021), it is possible that normative performance has been overestimated in our sample. It is also possible that unreported clinical (e.g. visual disorders) or other socio-demographic factors (e.g. first language, illiteracy) could limit performance on the task. We note in this regard that the UK Biobank is not representative of the UK population as a whole. Further investigations are required to determine best practice in both test design and threshold measurement that minimize false positives in typical adults. It is of course also important to minimise false negatives (i.e., missed diagnoses in those with PCA), though the current findings suggest a low rate of false negatives using our letter-based thresholds.

Further limitations of the current study arise from the relatively small sample of UCL participants diagnosed with PCA or typical AD and the restricted stimulus sets used herein. While we used a penalized likelihood logistic regression given the small PCA sample, estimates should be interpreted with caution and larger studies are required to validate the GILT across settings. Additionally, though our patient-based implementation of the GILT used the same stimulus sets and presentation as the UK Biobank, test administration was adjusted to allow for cognitive impairments. In particular, PCA and typical AD participants made verbal responses rather than using the touchscreen, and only discontinued testing when the cut-off thresholds were reached (rather than the timed limit in the UK Biobank). These time constraints may also have led to an underestimation of accuracy in the normative Biobank dataset. Further testing is required to determine whether GILT set and item (letter) effects play a significant role in performance, although these first release data in UK Biobank do not suggest material set effects. It will also be important to extend understanding of GILT performance outside the current UK Biobank, PCA and typical AD samples. Informing a GILT version for clinical use requires further testing involving larger pools of participants with visual and neurodegenerative conditions across ophthalmic and neurologic settings. Amendments for clinical testing might include adjusting administration (e.g. GILT-UKB responses are restricted to 12 items) and evaluating acceptability.

## Conclusions

The GILT allows the rapid measurement of cortical deficits in the recognition of visually degraded (incomplete) letters, a particular difficulty for individuals with PCA. The test can ordinarily be performed with a high level of degradation in both typical adults and those with a range of common ophthalmological disorders, as well as by participants with mild, typical AD. Clinical verification data suggest that the GILT has utility in detecting higher order cortical visual deficits characteristic of PCA while being insensitive to deficits arising from common age-related eye conditions (given that performance is not elevated in these latter cases). The GILT has a number of advantageous properties, including an increased test granularity compared to routine measures owing to the high number of letter completeness levels and the calculation of thresholds using letter-based scoring. This granularity offers increased opportunities both to detect early stage deficits and to track disease progression over time. In research contexts, salient opportunities afforded by the GILT include the ability to detect subtle cortical visual abnormalities at scale (given both its granularity and rapid measurement), in order to compare these abilities with candidate associated clinical, developmental and genetic factors. The ultimate clinical goals for this test include the potential to address knowledge gaps noted by eye and neurology professionals (Bowen et al., 2019) and to reduce diagnostic delays and misdiagnosis faced by individuals with dementia-related visual loss (Graff-Radford et al., 2021).

### Supplementary information

Below is the link to the electronic supplementary material.

Supplementary file1 (DOCX 319 KB)

## Data Availability

Data used in this work are available upon application to the UK BioBank (https://www.ukbiobank.ac.uk). Materials used in this work are available via UK BioBank (Data-Field 7669 ‘Resources’, https://biobank.ndph.ox.ac.uk/ukb/field.cgi?id=7669). The authors do not have permission to share data directly.

## Abbreviations

AD: Alzheimer’s disease
GILT: Graded Incomplete Letters Test
PCA: posterior cortical atrophy
UKB: UK Biobank
VOSP: Visual Object and Space Perception Battery

## Declarations

We thank all patient participants for their generous time and efforts supporting research. We are grateful to the UKBB Eye and Vision Consortium and Mr Stephen Bell and Dr Alan Young from the UKBB team for implementation of the test to the UKBB IT platform. In memory of Mrs Pam Southerden.

## Code availability

Code relevant to this work are available via GitHub (https://github.com/eccentricvision/IncompleteLetters). UK BioBank test instructions and stimuli are available under Data-Field 7669 ‘Resources’ (https://biobank.ndph.ox.ac.uk/ukb/field.cgi?id=7669).
