# Supplementary material for: The Graded Incomplete Letters Test (GILT): a rapid test to detect cortical visual loss, with UK Biobank implementation
Source: Behav Res Methods. 2024 Jun 18;56(7):7748–60. doi: 10.3758/s13428-024-02448-7 (PMC11362218; doi:10.3758/s13428-024-02448-7)
Supplement: Supplementary file 1 — Supplementary file1 (DOCX 319 KB) [file 13428_2024_2448_MOESM1_ESM.docx]

**GILT performance: percent correct**


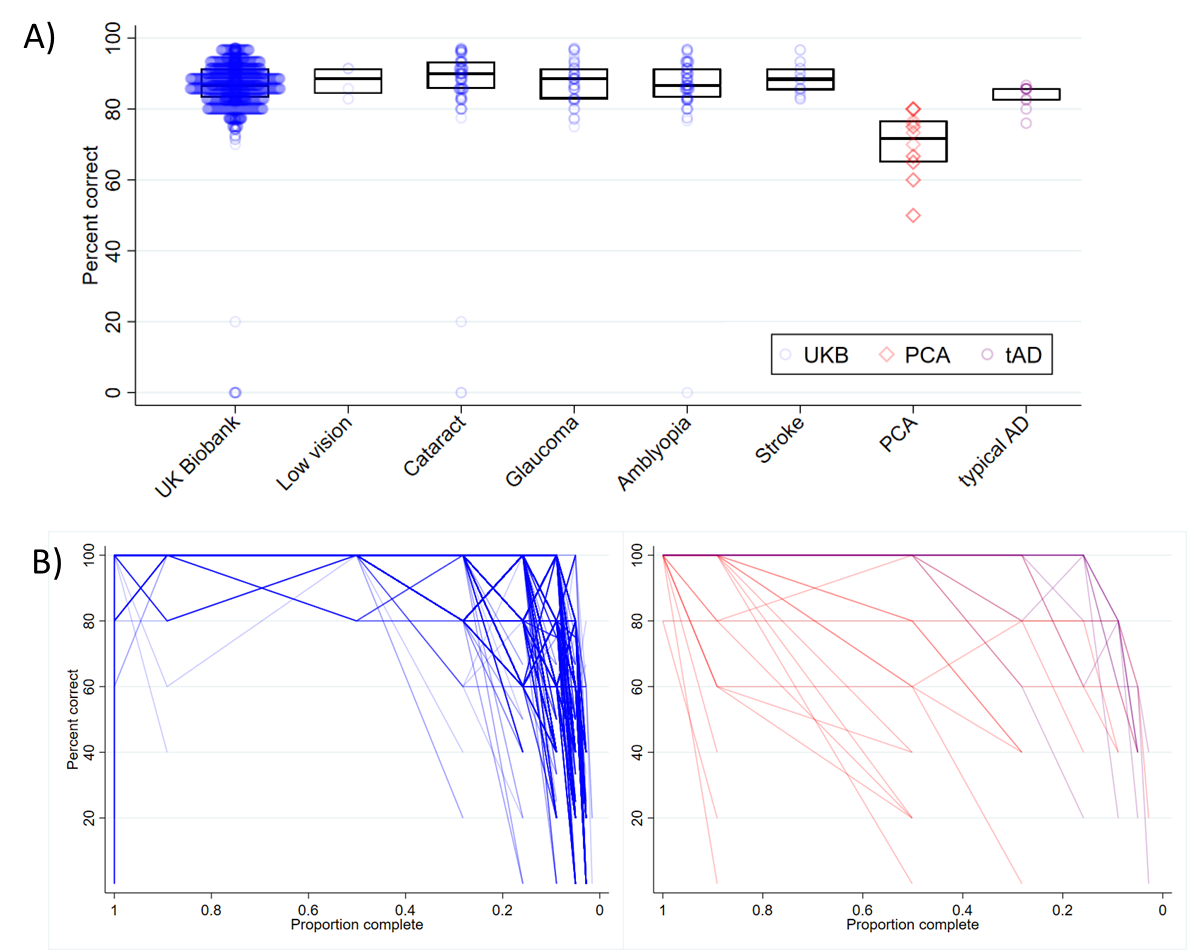


**Supplementary Figure 1** A) percent correct in UK Biobank without and with visual conditions or stroke, PCA and typical AD participants with group medians and interquartile ranges; B) relationship between percent correct and letter completeness in all UK Biobank (left), PCA and typical AD participants (right). For comparison purposes, data are only presented for participants who reached the 100% accuracy cut-off (i.e. making at least one error within the time limit).

In the total UKB sample, 10/2,359 (0.4%) participants performed at floor (0% accuracy). Of 10 participants performing at floor, two had cataract and one had amblyopia (with normal acuity). Of the seven remaining participants performing at floor, six provided sufficient (incorrect) responses to reach the next completeness level; the remaining participant ran out of time after only three trials. With the exception of participants performing at floor, UKB participants exhibited a tendency to make errors particularly from 0.28 completeness and below (Supplementary Figure 1B).

In the UCL sample, observed mean accuracy ranged from 50.0-80.0 in the PCA group and 76.0-86.7 in the typical AD group; notably, at the individual level, PCA or typical AD participants did not approach floor or ceiling performance. While all PCA and typical AD participants performed above floor, they consistently made errors earlier, in line with an overall exaggerated effect of completeness on their response accuracy (Supplementary Figure 1).

**GILT Primary outcome: completeness thresholds**


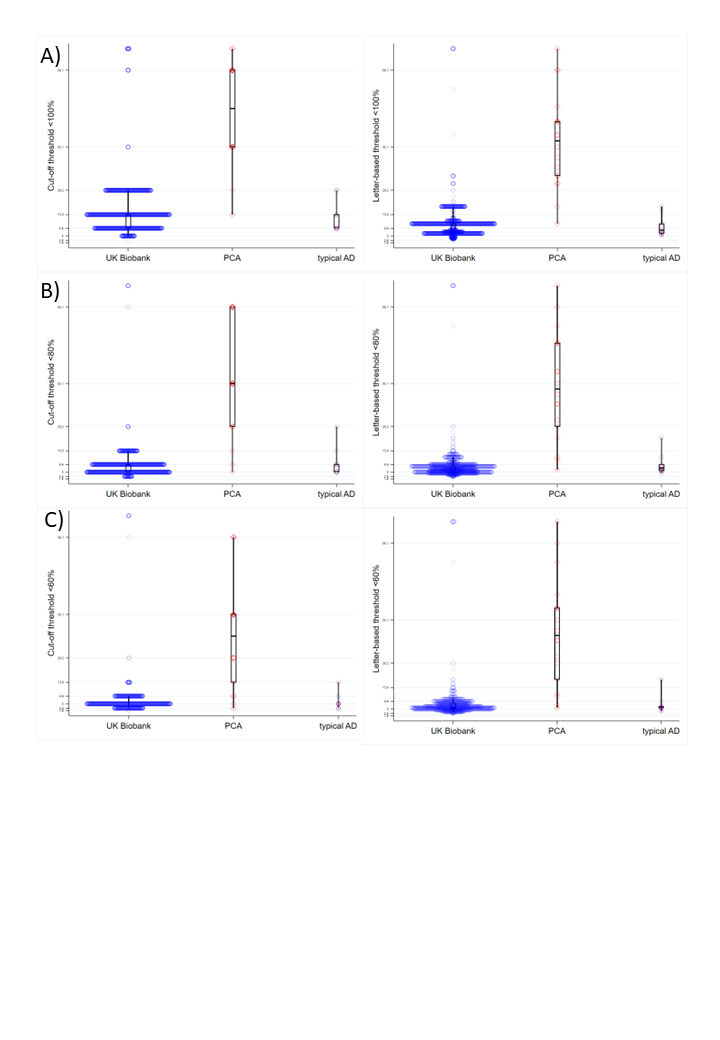


**Supplementary Figure 2** GILT completeness thresholds in UK Biobank participants without documented visual conditions or stroke, PCA and typical AD participants. Thresholds are defined on reaching accuracy cut-off of A) <100%, B) <80% or C) <60% accuracy (cut-off threshold; left); or with increasing score per correct letter (letter-based threshold; right).

**Supplementary Table 1.** Neuropsychological raw scores and estimated performance relative to normative datasets of patients with PCA and tAD.

|  | | *PCA (N=18)* | | *tAD (N=9)* | |  | |
| --- | --- | --- | --- | --- | --- | --- | --- |
| *Neuropsychology test* | *Max* | *Raw Score* | | | | *% patients below 5^th^%ile†* | |
|  |  | *PCA* | | *tAD* | | *PCA* | *tAD* |
| **Background Neuropsychology** | | | | | | | |
| Short Recognition Memory Test for words  (joint auditory/visual presentation) | 25 | 19.5 (16.8, 22.0) | | 23.0 (18.5, 23.5) | | 6/18 | 2/9 |
| Short Recognition Memory Test for faces  (visual presentation) | 25 | 17.0 (13.5, 20.5) | | 22.0 (21.5, 23.0) | | 8/17 | 1/9 |
| Concrete Synonyms test | 25 | 21.0 (19.0, 23.3) | | 20.0 (17.5, 24.0) | | 3/18 | 3/9 |
| Naming (verbal description) | 20 | 13.0 (7.5, 18.5) | | 15.0 (9.0, 18.0) | | 11/17 | 6/9 |
| Calculation (adapted GDA^b^) | 26 | 15.0 (10.5, 17.0) | | 15.0 (12.5, 18.0) | | 7/14 | 4/9 |
| Spelling (GDST^c^- Set B, first 20 items) | 20 | 11.0 (9.0, 17.0) | | 13.0 (9.0, 18.0) | | 1/17 | 0/9 |
| Reading (CORVIST^d^) | 16 | 16.0 (13.5, 16.0) | | 16.0 (16.0, 16.0) | | - | - |
| Gesture production test | 15 | 12.0 (11.0, 15.0) | | 15.0 (15.0, 15.0) | | - | - |
| Digit span (forwards) | 12 | 7.0 (6.0, 8.5) | | 7.0 (5.5, 8.5) | | 1/17 | 1/9 |
| Digit span (backwards) | 12 | 3.0 (2.0, 5.0) | | 5.0 (2.0, 6.5) | | 3/17 | 3/9 |
|  |  |  | |  | |  |  |
| ***Visual Assessment*** | | | | | | | |
| *Early visual* |  |  |  | |  |  | |
| Visual acuity (CORVIST^d^): Snellen (median) | 6/9 | 6/9 | | 6/9 | | - | - |
| Figure-ground discrimination (VOSP^e^) | 20 | 16.0 (13.5, 17.0) | | 20.0 (19.0, 20.0) | | 15/18 | 1/9 |
| Shape discrimination^f^ | 20 | 15.5 (12.0, 18.5) | | 20.0 (19.0, 20.0) | | Healthy participants do not make errors | |
| Hue discrimination (CORVIST^d^) | 4 | 3.5 (3.0, 4.0) | | 4.0 (4.0, 4.0) | |  |  |
|  |  |  |  | |  |  | |
| *Visuoperceptual* |  |  |  | |  |  | |
| Fragmented letters (VOSP) | 20 | 3.5 (1.0, 8.0) | | 18.0 (15.5, 19.5) | | 17/18 | 2/9 |
| Object Decision (VOSP) | 20 | 9.5 (6.0, 11.3) | | 18.0 (15.0, 18.5) | | 12/14 | 1/9 |
| Unusual and usual views: Unusual | 20 | 3.0 (1.0, 4.0) | | 11.0 (6.0, 14.5) | | 13/13 | 5/9 |
| Usual | 20 | 12.0 (9.0, 18.0) | | 20.0 (18.5, 20.0) | | 13/13 | 4/9 |
|  |  |  |  | |  |  | |
| *Visuospatial* |  |  |  | |  |  | |
| Dot counting (VOSP) | 10 | 2.5 (0.0, 8.3) | | 9.0 (8.0, 10.0) | | 14/18 | 3/9 |
| Number location (VOSP) | 10 | 4.0 (0.5, 6.0) | | 7.0 (3.0, 9.0) | | 10/13 | 4/9 |
| A Cancellation: Completion time | 90s | 61.0 (49.0, 87.0) | | 30.0 (22.5, 34.0) | | 14/15 | 4/9 |
| A Cancellation: Number of letters missed | 19 | 3.0 (1.0, 9.0) | | 0.0 (0.0, 0.0) | | - | - |

Medians and interquartile ranges are reported for neuropsychological raw scores.

*†* individual missing items are indicated in the denominator. Measures are adopted from a standard published cognitive battery (Firth et al., 2019; Yong et al., 2014).

^a^Mini-Mental State Examination. ^b^Adapted Graded Difficulty Arithmetic test. ^c^Graded Difficulty Spelling Test. ^d^Cortical Visual Screening Test. ^e^Visual Object and Space Perception Battery.

**Supplementary Table 2.** Associations between PCA diagnosis and GILT impairment, with comparison to UKB either without (left) or with documented visual conditions (right). GILT impairment is defined using a standard cut-off (<5^th^ percentile in UKB without visual conditions reaching A) 100% or B) 60% accuracy) using letter-based thresholds at A) 100% accuracy cut-off or B) 60% accuracy cut-off. Sensitivity, specificity, positive predictive and negative predictive values (PPV; NPV) are presented. UKB participants with stroke have been excluded.

|  | A) Letter-based threshold: 100% accuracy cut-off | | | | |
| --- | --- | --- | --- | --- | --- |
|  | PCA diagnosis vs UKB (no visual dx) | |  | PCA diagnosis vs UKB (visual dx) | |
|  | Positive | Negative |  | Positive | Negative |
| Positive | 16 | 32 | Positive | 16 | 5 |
| Negative | 2 | 2010 | Negative | 2 | 233 |
|  |  |  |  |  |  |
| Sensitivity | 88.9 | | Sensitivity | 88.9 | |
| Specificity | 98.4 | | Specificity | 97.9 | |
| PPV | 33.3 | | PPV | 76.2 | |
| NPV | 99.9 | | NPV | 99.1 | |
|  |  |  |  |  |  |

|  | B) Letter-based threshold: 60% accuracy cut-off | | | | |
| --- | --- | --- | --- | --- | --- |
|  | PCA diagnosis vs UKB (no visual dx) | |  | PCA diagnosis vs UKB (visual dx) | |
|  | Positive | Negative |  | Positive | Negative |
| Positive | 16 | 56 | Positive | 16 | 7 |
| Negative | 2 | 1227 | Negative | 2 | 119 |
|  |  |  |  |  |  |
| Sensitivity | 88.9 | | Sensitivity | 88.9 | |
| Specificity | 95.6 | | Specificity | 94.4 | |
| PPV | 22.2 | | PPV | 69.6 | |
| NPV | 99.8 | | NPV | 98.3 | |
|  |  |  |  |  |  |
